# Structure basis of neutralization by a novel site II/IV antibody against respiratory syncytial virus fusion protein

**DOI:** 10.1371/journal.pone.0210749

**Published:** 2019-02-07

**Authors:** Qingqing Xie, Zhao Wang, Fengyun Ni, Xiaorui Chen, Jianpeng Ma, Nita Patel, Hanxin Lu, Ye Liu, Jing-Hui Tian, David Flyer, Michael J. Massare, Larry Ellingsworth, Gregory Glenn, Gale Smith, Qinghua Wang

**Affiliations:** 1 Department of Bioengineering, Rice University, Houston, Texas, United States of America; 2 Verna and Marrs McLean Department of Biochemistry and Molecular Biology, Baylor College of Medicine, Houston, Texas, United States of America; 3 Novavax, Inc., Gaithersburg, Maryland, United States of America; University of Iowa, UNITED STATES

## Abstract

Globally, human respiratory syncytial virus (RSV) is a leading cause of lower respiratory tract infections in newborns, young children, and the elderly for which there is no vaccine. The RSV fusion (F) glycoprotein is a major target for vaccine development. Here, we describe a novel monoclonal antibody (designated as R4.C6) that recognizes both pre-fusion and post-fusion RSV F, and binds with nanomole affinity to a unique neutralizing site comprised of antigenic sites II and IV on the globular head. A 3.9 Å-resolution structure of RSV F-R4.C6 Fab complex was obtained by single particle cryo-electron microscopy and 3D reconstruction. The structure unraveled detailed interactions of R4.C6 with antigenic site II on one protomer and site IV on a neighboring protomer of post-fusion RSV F protein. These findings significantly further our understanding of the antigenic complexity of the F protein and provide new insights into RSV vaccine design.

## Introduction

Human respiratory syncytial virus (RSV) is a leading cause of pneumonia and bronchiolitis in premature newborns, young children, and adults over 65 years old. In the United States, RSV is responsible for over 2 million outpatient visits and nearly 60,000 hospitalizations in children under 5 years of age and 11,000–17,000 deaths in adults each year [1–3]. Globally, RSV is responsible for over 30 million lower respiratory tract infections (LRTI), 3 million hospitalizations, and 50,000–75,000 deaths each year in children under 5 years with the majority of incidents in developing countries [4–6]. To date, there is no approved RSV vaccine, and palivizumab (Synagis) is the only passive prophylaxis approved for prevention of RSV infection in newborns under 24 months of age. However, the high cost and 5-dose treatment regimen prevent the use and availability of palivizumab in developing countries [7–9].

Human RSV is an enveloped RNA virus and a member of the *Pneumoviridae* family that is comprised of RSV/A and RSV/B subgroups [10–15]. The RSV genome is a negative-stranded 15 kilobase RNA that encodes 11 structural and non-structural proteins. Three structural proteins are found on the virus surface: the small hydrophobic (SH) protein is a pentameric ion channel; the attachment (G) glycoprotein mediates attachment of the virus particle to bronchial epithelium; and the fusion (F) glycoprotein mediates fusion of the viral envelope with the host membrane, permitting delivery of the viral genome into the cell [16, 17].

The G and F glycoproteins are the targets of host immune response. G protein is heavily glycosylated with >60% of its 90 kilo-dalton (kD) mass comprised of carbohydrates. G glycoproteins are heterogeneous with limited sequence homology (53%) and little antigenic cross-reactivity between the two subgroups [18–21]. In sharp contrast, F glycoprotein sequences are well conserved (>90%) with a high degree of antigenic cross-reactivity between the subgroups [22]. Consequently, F glycoprotein has been the major target for vaccine development.

Human RSV F glycoprotein is initially synthesized as a single-chain 574-residue polypeptide (F0) with a molecular weight of ~70 kD. F0 contains two furin-like cleavage sites at residues 109 and 136. Cleavage of both furin sites on F0 is required for its infectivity [23]. The double cleavage results in the removal of the intervening 27-residue peptide (p27 peptide), generating the N-terminal F2 fragment (~20 kD) and the larger C-terminal F1 fragment (~48 kD) that are covalently linked through two disulfide bonds. The F1 fragment harbors the hydrophobic fusion peptide (FP) at the N-terminus, and the hydrophobic transmembrane domain (TM) and cytoplasmic tail (CT) at the C-terminus. The F2 fragment has two glycans at residues N27 and N70 and the F1 fragment has a single glycan at residue N500. The F2-F1 subunit self-associates to form trimers which are anchored on the virus envelope *via* the TM domain on F1. Accompanying the virus infection cycle, the F glycoprotein undergoes significant unidirectional rearrangement from the pre-fusion conformation to a stable post-fusion conformation, facilitating fusion of the viral envelope with the host membrane [17].

RSV virus neutralizing antibodies were first reported over 30 years ago [24] and are widely believed to correlate with protection against severe LRTI [25]. The humanized monoclonal antibody (mAb) palivizumab is currently the only immune prophylaxis approved for treatment of newborns at elevated risk of severe infection [8, 26]. Since then, the crystal structures of the F glycoprotein in pre-fusion and post-fusion conformations, in complex with murine and human mAbs, have resulted in the identification and fine mapping of a number of antigenic epitopes [27, 28]. Antigenic site II is located on the F1 fragment spanning amino-acid residues 254–278 that is further classified into antigenic site IIa recognized by neutralizing mAbs palivizumab and hRSV14N4, and antigenic site IIb targeted by motavizumab and hRSV3J20 [29, 30]. However, given the fact that motavizumab is an affinity matured variant of palivizumab [31], the detailed differences of their epitopes cannot be delineated without high-resolution structures of these mAbs with RSV F. In addition, non-neutralizing human mAbs (4B6, 9J5 and 12I1) bind to antigenic site II at a position that is distinct from palivizumab and motavizumab. This non-neutralizing epitope has been designated as antigenic site VII [30]. Antigenic site IV is another neutralizing epitope also located on the F1 encompassing residues 422–471 [27]. Site IV neutralizing mAbs include 101F [29, 32, 33] and humanized RSHZ19 [34]. Sites II, IV and VII epitopes are conserved between RSV subgroups and are present on both the pre-fusion and post-fusion conformations [33, 35]. Potent neutralizing epitopes have also been identified and mapped on the RSV F pre-fusion conformation. Antigenic site zero (Ø) is the target of human mAbs D25 and AM22 [36] and murine 5C4 [17]. Site Ø epitope is conserved within the RSV/A subgroup and less conserved within the RSV/B subgroup [14].

Here we present the generation and characterization of a number of new murine mAbs elicited using RSV F nanoparticles (NP). Intriguingly, we identified a novel mAb, designated as R4.C6, which recognizes both pre-fusion and post-fusion RSV F, and binds with nanomole affinity to a previously unknown neutralizing epitope located at an intermediate position between antigenic sites II and IV in the F1 fragment. Single particle cryo-electron microscopy (cryo-EM) and 3D reconstruction were used to determine the fragment antigen-binding region (Fab) of R4.C6 in complex with post-fusion RSV F glycoprotein at 3.9 Å resolution. The critical structural insights of the novel neutralizing epitope recognized by R4.C6 may inform vaccine development and lend understanding to the progressive structural changes that the F protein undergoes during the process of RSV infection.

## Results

### Generation and characterization of mAbs

RSV F-specific mAbs were generated using modified standard methods [37] in BALB/c mice immunized with RSV F NP [38]. Four hybridomas producing RSV F specific mAbs were selected for characterization. The mAbs were designated as R6.29 and R1.42 (IgG1 subclass), R4.C6 IgG2a, and R6.46 IgG3 (Table 1).

**Table 1:** Characterization of RSV F-specific mAbs.

RSV F mAb	IgG subclass/ light chain	RSV F antigenic site	RSV/A Long neutralization (IC_50_, ng/mL)	Binding Affinity (*K*_D_, nM)	
				Site II peptide^a^	RSV F NP^b^
**mAb isolated in this study**					
R4.C6	IgG2a/к	Site II/IV	1078	12.5	0.07
R6.46	IgG3/к	Site IIa	727	442	3.21
R1.42	IgG1/к	Site IV	19.6	No binding	0.04
R6.29	IgG1/к	Site VII	No^c^	No binding	1.59
**Control mAbs**					
Palivizumab	IgG1/к	Site IIa	323	370	0.51
Motavizumab	IgG1/к	Site IIb	20.8	26.7	0.04

^a^Antigenic site II synthetic peptide of RSV F (254-NSELLSLINDMPITNDQKKLMSNNV-278)

^b^RSV F NP: RSV F glycoprotein nanoparticles

^c^Indicates poor or no neutralization of RSV/A Long (IC_50_ >10 μg/mL)

Antibody binding affinities with RSV F NP and the antigenic site II peptide (residues 254–278) were determined by surface plasmon resonance (SPR) using a Biacore T200 instrument. R4.C6 bound RSV F NP with *K*_D_ = 0.07 nM, which was 7-fold higher than palivizumab (*K*_D_ = 0.51 nM) and comparable to motavizumab (*K*_D_ = 0.04 nM). R4.C6 bound the site II peptide (residues 254–278) with *K*_D_ = 12.5 nM, which was 30-fold greater than palivizumab (*K*_D_ = 370 nM) and comparable to motavizumab (*K*_D_ = 26.7 nM). In addition, R6.46, R6.29, and R1.42 bound RSV F NP with *K*_D_ of 0.04–3.21 nM. R6.46 also bound the site II peptide (*K*_D_ = 442 nM) while R1.42 and R6.29 failed to bind (Table 1, S1 Fig).

Virus neutralization assay was used to determine the neutralization potency of these mAbs. R4.C6 and R6.46 neutralized RSV/A Long with IC_50_ = 1078 and 727 ng/mL, respectively, which were slightly higher than that of palivizumab (IC_50_ = 323 ng/mL). MAb R1.42 had the strongest neutralization potency of all the mAbs newly identified in this study (IC_50_ = 19.6 ng/mL), which was 15-fold greater than that of palivizumab and similar to motavizumab (IC_50_ = 20.8 ng/mL). R6.29 failed to neutralize RSV/A Long (IC_50_ >10 μg/mL) (Table 1).

### Epitope binning of mAbs by biolayer interferometry (BLI)

To further characterize the antigenic sites bound by these mAbs, epitope binning was performed by BLI using an Octet QK384 system. Histidine tagged RSV F710 post-fusion was immobilized on anti-penta-histidine biosensor tips. Captured RSV F710 was exposed to individual mAbs in two steps to determine the competition. Palivizumab and RSV14N4 (site IIa), motatvizumab and RSV3J20 (site IIb), and RSHZ19 (site IV) were used as controls to define the different antigenic sites.

**Fig 1:**
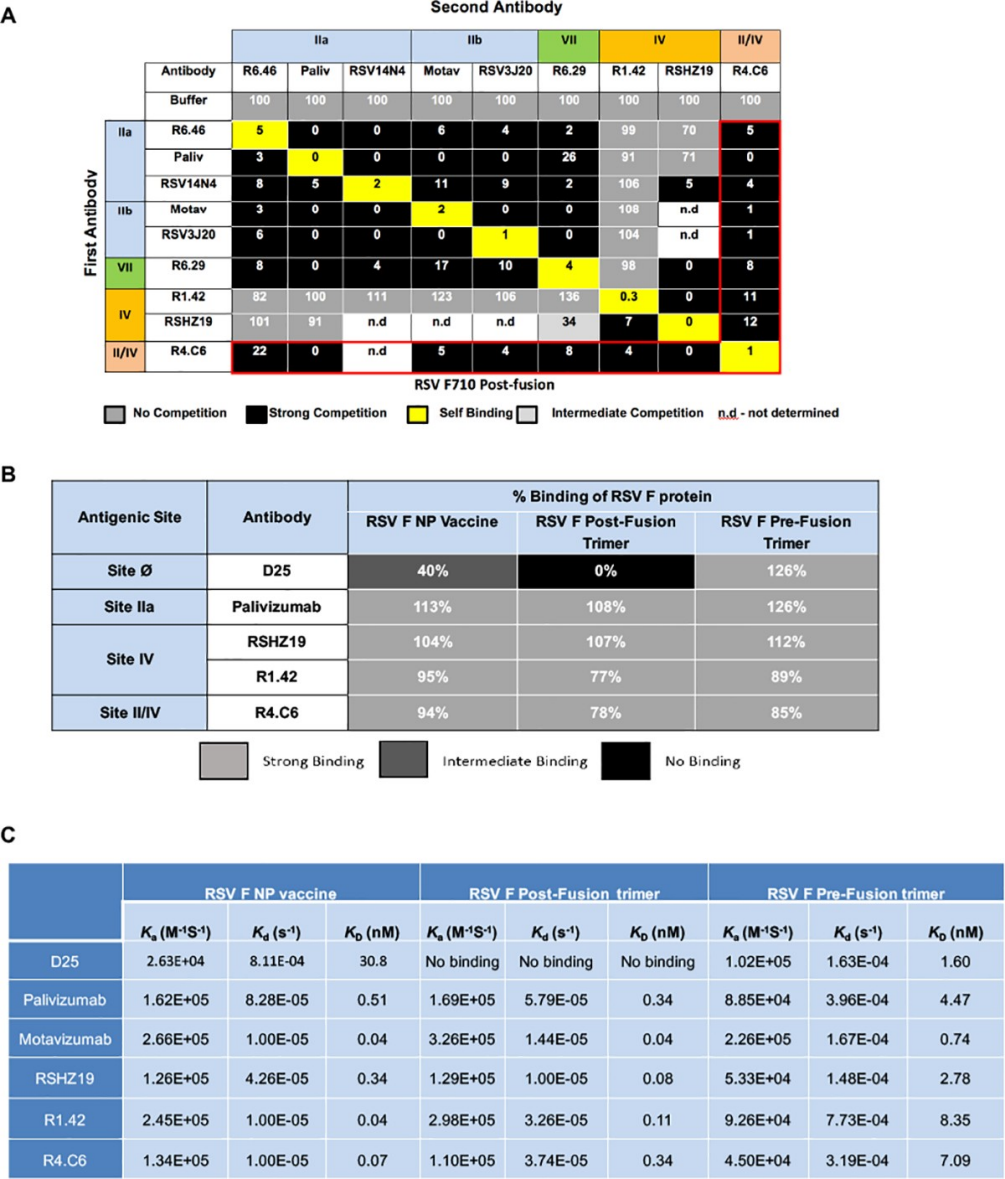
Epitope binning and binding of mAbs to RSV F glycoprotein. (A) Epitope binning for mAbs binding to RSF F710 post-fusion. Antibody cross-competition was determined by BLI using an Octet QK384 system. Histidine tagged RSV F710 post-fusion protein was immobilized on anti-penta-histidine biosensor tips. The data indicate the percent binding of the competing second antibody in the presence of the first antibody, compared with the competing antibody alone. Black cells indicate complete competition (percent binding between 0% and 33%), light gray cells indicate intermediate completion (percent binding between 34% and 60%), and dark gray cells indicate no competition (percent binding >60%). Yellow cells indicate self competition. Antigenic sites IIa/IIb, VII, IV and II/IV are indicated at top and side colored cells. Cells outlined in red indicate R4.C6 competition with antigenic site-specific control antibodies palivizumab and RSV14N4 (site IIa), motavizumab and RSV3J20 (site IIb), R6.29 (site VII), R1.42 and RSVZ19 (site IV). Palivizumab (Pali), motavizumab (Mota), not determined (n.d.). (B) Binding of mAbs to RSV F NP, post-fusion and pre-fusion. Percent (%) binding was determined by BLI using an Octet QK384 system, referred to binding of RSV F mAbs with RSV F NP, post-fusion F or pre-fusion F. Light grey cells indicate strong binding, dark grey cells indicate intermediate binding, and black cells indicate no binding. (C) Binding kinetics of mAbs to RSV F NP, post-fusion and pre-fusion. K_D_: Apparent equilibrium dissociation constant calculated as *k*_d_/*k*_a_; *k*_a_: association rate from the association curves; *k*_d_: dissociation rate from the dissociation curves. Also see S2 Fig for sensorgrams.

Antibodies R6.29 and R6.46 both competed the binding of site IIa and site IIb-specific antibodies (Fig 1A). Although both antibodies were site II specific with similar binding affinities for RSV F NP (*K*_D_ of 1.59 nM and 3.21 nM, respectively), R6.46 was found to neutralize RSV/A Long and bound to site II peptide while R6.29 had no measurable neutralization activity and did not bind to site II peptide (Table 1), suggesting these mAbs bound site II differently. The properties of R6.29 were similar to non-neutralizing site II-specific antibodies, the epitope of which was recently designated as antigenic site VII [30]. The relatively weaker neutralization activity of R6.46 made it similar to palivizumab (site IIa). On the other hand, R1.42 competed the binding of RSVZ19, indicating these antibodies target antigenic site IV (Fig 1A).

Interestingly, R4.C6 broadly competed the binding of antibodies targeting antigenic site IIa (palivizumab, RSV14N4 and R6.46), site IIb (motavizumab and RSV3J20), site IV (RSHZ19 and R1.42) and non-neutralizing site VII (R6.29) (Fig 1A). These results suggested that R4.C6 uniquely recognizes a previously undefined epitope spanning neutralizing antigenic sites IIa/b and IV, and non-neutralizing site VII.

Antigenic site II and site IV are present in both pre- and post-fusion RSV F. Using BLI-based Octet QK384 system, we compared the interactions of various RSV F conformers with a panel of mAbs (Fig 1B). D25 (site Ø-specific antibody) [17] had intermediate binding for the RSV F NP, no binding with post-fusion RSV F and strong binding with pre-fusion RSV F (Fig 1B). On the other hand, palivizumab, RSHZ19, R4.C6 and R1.42 had similar binding with all three RSV F conformers, even though the binding percentages of R4.C6 and R1.42 were slightly lower than those of palivizumab and RSHZ19 (Fig 1B).

We further determined the binding kinetics of a panel of mAbs (D25, palivizumab, motavizumab, RSHZ19, R1.42 and R4.C6) with various RSV F conformers by SPR (Fig 1C, S2 Fig). Consistent with the BLI binding results in Fig 1B, D25 interacted very weakly with RSV F NP vaccine, did not bind to post-fusion RSV F, and bound to pre-fusion RSV F tightly (*K*_D_ = 1.60 nM). All other mAbs including R4.C6 exhibited a narrow range of *K*_D_ in binding to RSV F NP (0.04~0.51 nM), to post-fusion RSV F (0.04~0.34 nM) and to pre-fusion RSV F (0.74~8.35 nM).

### Structure of R4.C6 in complex with RSV F710 in post-fusion state

**Fig 2:**
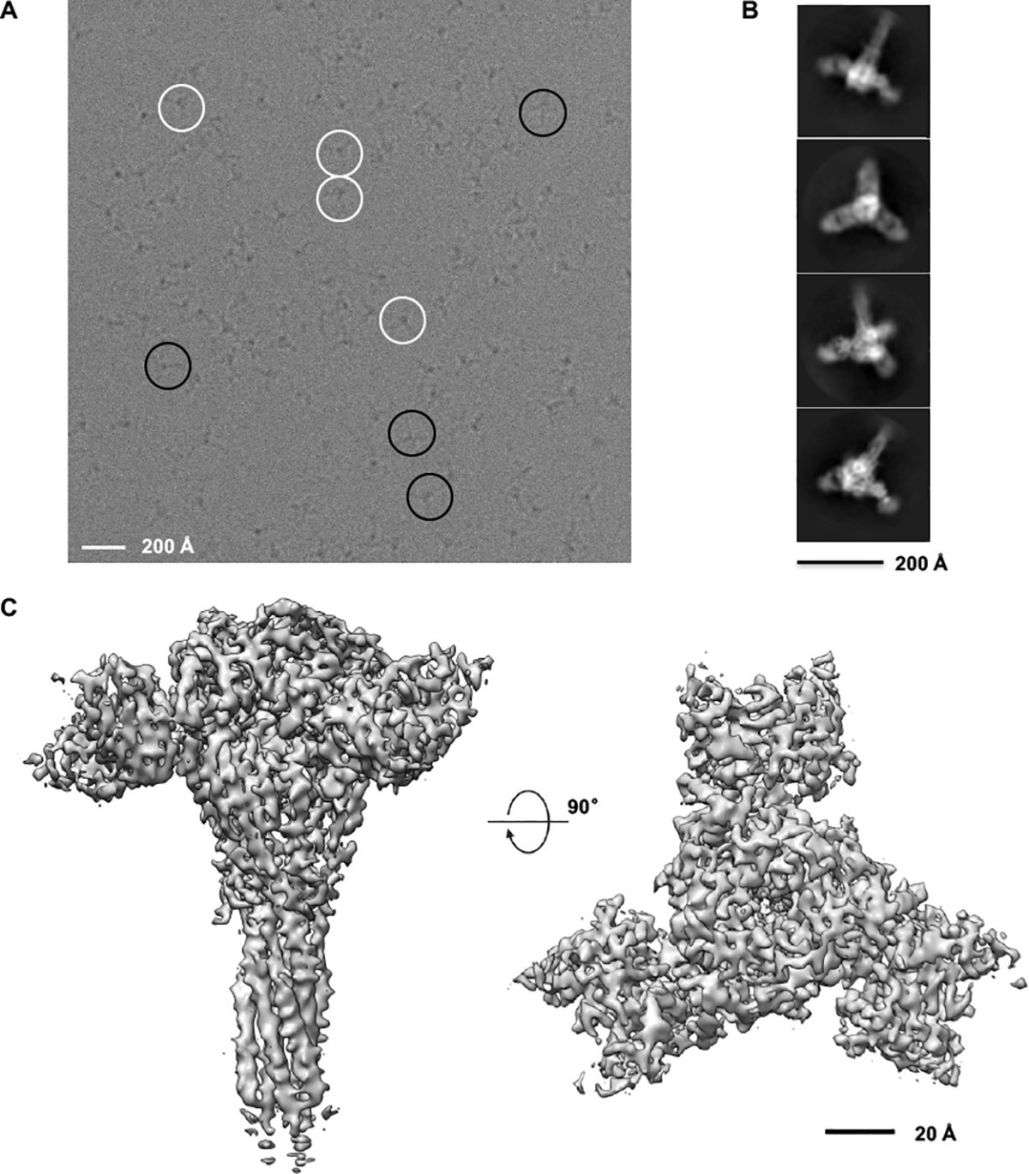
Cryo-EM of the purified post-fusion RSV F710 in complex with R4.C6 Fab. (A) Representative raw electron image of ice-embedded RSV F-R4.C6 complex on graphene oxide grids recorded using K2 Summit camera. White circles indicate particles with top view; black circles indicate particles with side view. Scale bar, 200 Å. The shown image is motion-corrected using MotionCor2. (B) Representative 2D class averages of the RSV F-R4.C6 Fab complex obtained by using RELION2.0. C3 symmetry is apparent in these 2D class averages. (C) Cryo-EM 3D map of R4.C6 Fv in complex with RSV F shown as side view (Left) and top view (Right). Also see S4 Fig for cryo-EM 3D reconstruction.

Because of the unique binding properties of R4.C6 with antigenic sites II and IV, we next focused on determining the structural basis of this antibody with RSV F. Purified RSV F710-R4.C6 Fab complex (S3 Fig) was used for single-particle cryo-EM analysis (Fig 2). Fig 2A shows a representative raw image of ice-embedded RSV F-R4.C6 complex on graphene oxide grids recorded using K2 Summit camera, and Fig 2B displays representative 2D class averages of the complex obtained by using RELION2.0. Totally, we used 234,479 particles in the final 3D reconstruction, and obtained a final map ranging from 2.7 to 4.6 Å, with an overall 3.9 Å resolution according to the gold standard Fourier shell correlation (FSC) at 0.143 (S1 Table, S4 Fig).

At current resolution, we can confidently recognize one RSV F trimer and three copies of the R4.C6 molecules (Fig 2C). The crystal structures of RSV F in pre-fusion or post-fusion conformation (PDB codes: 4JHW and 3RRR) [17, 32] were individually docked into the cryo-EM map by using Chimera [39], where the post-fusion structure produced a much better match. As for the Fab portion, an Fv model comprising known structures of heavy chain (HC) and light chain (LC) variable domains with the highest sequence homology to R4.C6 was used. The constant region of the three R4.C6 Fab molecules was not modeled due to the relatively poor electron density. The model of the RSV F-R4.C6 complex was refined against the cryo-EM map using Real-space-refine in Phenix [40], followed by manually adjustment using Coot [41], and finally PCST refinement to improve the geometry (S1 Table). The overall quality of the map allowed the resolvability of the majority of side chains (S5 Fig).

**Fig 3:**
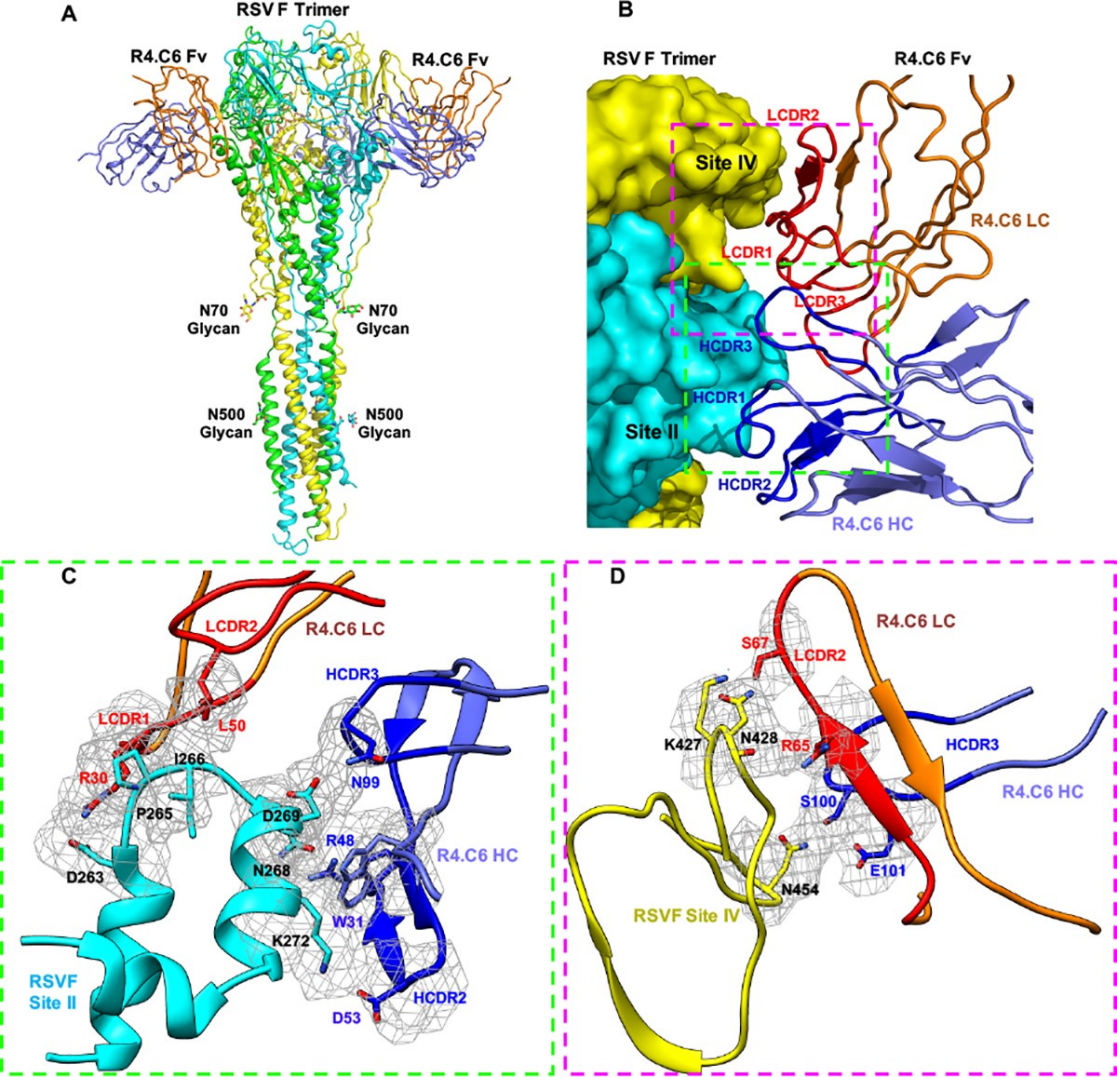
Structure of post-fusion RSV F710 in complex with R4.C6. (A) Ribbon representation of the model of R4.C6 Fv bound to RSV F glycoprotein trimer in post-fusion conformation in side view. Each protomer of RSV F has a different color (cyan, yellow, and green). Only two of the three R4.C6 Fv molecules (light chain in orange color and heavy chain in blue color) are shown for clarity. The N-acetyl-D-glucosamine moieties attached to N70 and N500 are shown. (B) The interface between RSV F and R4.C6 heavy chain (HC, blue color) and light chain (LC, orange color). CDRs are labeled for R4.C6 Fv (HC, dark blue color; LC, red color). Antigenic site II and IV are also labeled. Two regions to be zoomed in (C) and (D) are highlighted by dashed squares. (C) Detailed interactions between R4.C6 and RSV F antigenic site II. (D) Detailed interactions between R4.C6 and RSV F antigenic site IV. RSV F site II is colored as cyan and site IV is colored as yellow. Cryo-EM maps around the residues in close proximity at the RSV F-R4.C6 interface are shown in grey mesh. The same color scheme is used in (B~D).

The final RSV F-R4.C6 structural model has a RSV F trimer surrounded by the Fv domains of three R4.C6 Fab molecules (Fig 3A). The central RSV F has three protomers with each protomer composed of F2 and F1 that are covalently linked by two disulfide bonds. The N- and C-terminal domains of F1 in each protomer pack together to form a six-helical bundle, a key characteristic shared by many membrane-fusion proteins in post-fusion state [42].

### Epitope mapping of R4.C6

Cryo-EM map at the interface between RSV F trimer and each R4.C6 was well defined to allow confident identification of the epitope (Fig 3B–3D). Each R4.C6 recognizes a quaternary epitope on RSV F that consists of antigenic site II on one protomer, and antigenic site IV on another protomer (Fig 3B). Four complementarity determining regions (CDRs) of R4.C6 are involved in interacting with RSV F, which resulted in the burial of about 1571 A^2^ surface area between them.

The heavy chain HCDR2 and HCDR3 and light chain LCDR1 and LCDR2 of R4.C6 interact with antigenic site II. At the interface, residues D53 on R4.C6 HCDR2 and K272 on RSV F, N99 on R4.C6 HCDR3 and D269 on RSV F, R48 on R4.C6 HCDR2 and N268 on RSV F, W31 on R4.C6 HC and D269/K272 on RSV F are found in close proximity. Furthermore, R30 on R4.C6 LCDR1 and D263 on RSV F, L50 on R4.C6 LCDR2 and the loop formed by RSV F P265 and I266 are in close contact (Fig 3C). These detailed interactions between RSV F site II at residues 263–272 and R4.C6 revealed by our cryo-EM structure are consistent with the SPR analysis using site II linear peptides corresponding to RSV F residues 254-NSELLSLINDMPITNDQKKLMSNNV-278 and its various N-terminal and/or C-terminal truncations (Table 2, S6 Fig). R4.C6 bound with the full-length peptide at *K*_D_ of 12.5 nM and at reduced affinities with peptides truncated by 6 or 10 residues from the N-terminus. Furthermore, R4.C6 failed to bind when the C-terminal 6 or 10 residues were deleted (Table 2). Interestingly, R4.C6 did not bind to the peptide containing residues 254–273, despite the presence of residues 263–272 (Table 2). This may be due to a disruption of the α-helix on which N268, D269 and K272 reside, leading to the loss of optimal presentation of these residues to interact with R4.C6 HC (Fig 3C).

**Table 2:** Binding of R4.C6 to site II synthetic peptides determined by surface plasmon resonance.

Antigenic site II peptides	Binding affinity (*K*_D_, nM)	
	R4.C6	Palivizumab
254-NSELLSLINDMPITNDQKKLMSNNV-278	12.5	370
254-NSELLSLINDMPITNDQKKL-273	No binding	No binding
260-LINDMPITNDQKKLMSNNV-278	84.2	No binding
254-NSELLSLINDMPITN-268	No binding	No binding
259-SLINDMPITNDQKKL-273	No binding	No binding
264-MPITNDQKKLMSNNV-278	110	No binding

Moreover, the R4.C6 LCDR2 and HCDR3 are in close contact with RSV F antigenic site IV spanning residues 422–471 on a neighboring protomer (Fig 3D), involving residues S67 on R4.C6 LCDR2 with K427 and N428 on RSV F, R65 on R4.C6 LCDR2 with N428 on RSV F, and S100 and E101 on R4.C6 HCDR3 with N454 on RSV F. Although we were unable to directly verify the contributions of site IV to binding to R4.C6 by additional means, multiple lines of evidence strongly support its roles in this regard: (1) the epitope binning data in Fig 1A clearly demonstrated direct competition between R4.C6 and site IV-binding mAbs such as R1.42 and RSHZ19; (2) the direct inter-molecular interactions between site IV and R4.C6 in the cryo-EM structure that we just described; and (3) more importantly, R4.C6 binds to post-fusion F at *K*_D_ of 0.34 nM (Fig 1C), which is about 30-fold stronger than its binding to site II peptide alone (*K*_D_ of 12.5 nM) (Table 1).

### Comparison of site II and site IV mAbs with known structures

**Fig 4:**
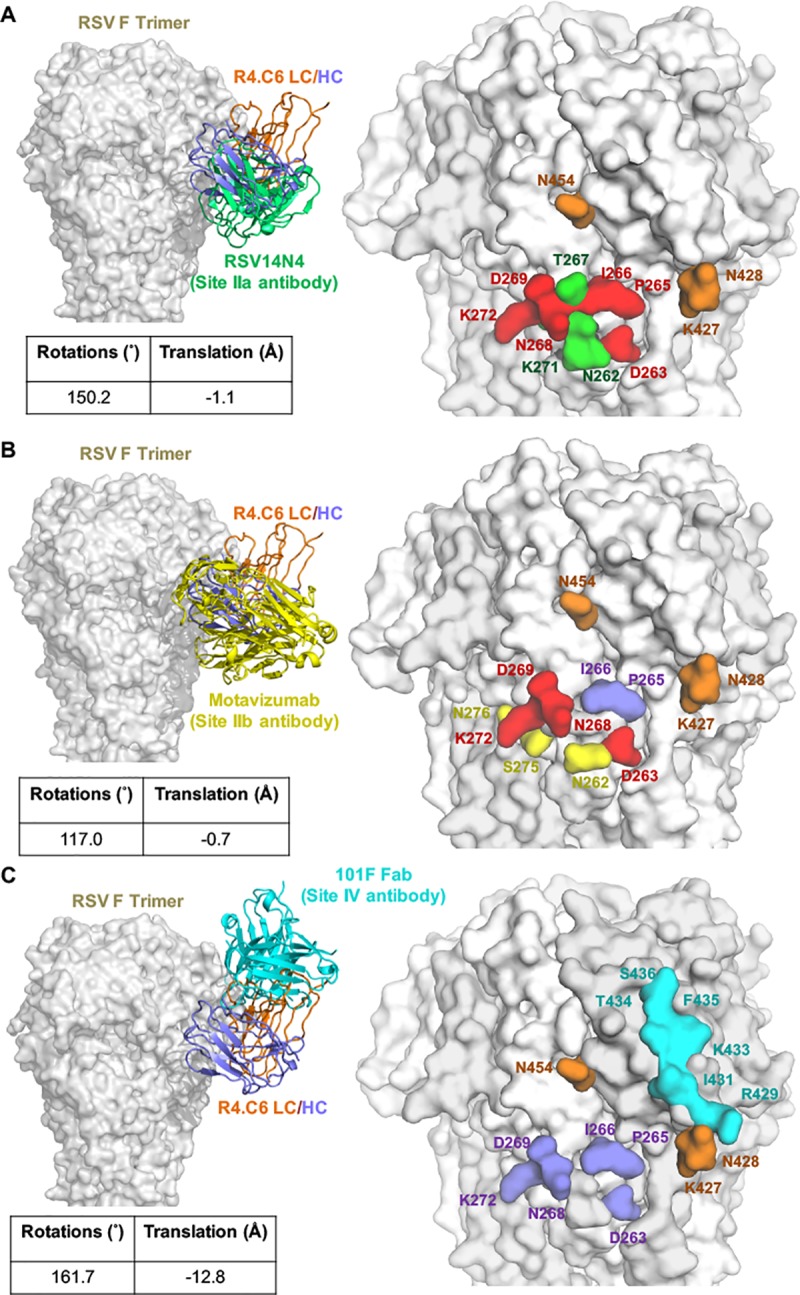
Comparison of known structures of post-fusion RSV F and site II or site IV antibodies. **(**A) Comparison of the structure of RSV F-R4.C6 with RSV14N4 bound to site II of RSV F (PDB code 5J3D). Left: RSV F is shown as surface presentation, R4.C6 in ribbons presentation colored in orange (light chain) and blue (heavy chain), and RSV14N4 in ribbons colored in green. Right: Overlap of R4.C6 and RSV14N4 epitopes. Shared residues between the two epitopes are colored red. Unique residues for RSV14N4 are colored as green. (B) Comparison of the structure of RSV F-R4.C6 with motavizumab–site II peptide complex (PDB code 3IXT). Left: Motavizumab in ribbons colored in yellow. Right: Overlap of R4.C6 and motavizumab epitopes. Shared residues between the two epitopes are colored red. Unique residues for motavizumab are colored as yellow. (C) Comparison of the structure of RSV F-R4.C6 with Fab 101F–site IV peptide complex (PDB code 3O45). Left: Fab 101F is shown in ribbons colored in cyan. Right: Overlap of R4.C6 and Fab 101F epitopes. Unique residues at site IV for Fab 101F are colored as cyan. In all panels, unique residues for R4.C6 are colored as blue for site II and orange for site IV.

The antigenic site II of RSV F is divided into antigenic site IIa and IIb for neutralizing poses, and site VII for non-neutralizing poses [30]. There are two known structures of RSV F site II-specific antibodies: hRSV14N4 binds to antigenic site IIa of post-fusion RSV F (PDB code: 5J3D) [30], and motavizumab binds a peptide from antigenic site IIb (PDB code: 3IXT) [43]. Even though both antibodies target antigenic site II, motavizumab binds RSV F at an angle that is 42° away from the binding angle of hRSV14N4 [30]. By superimposing the antigenic site II residues N254 to N277 of hRSV14N4-RSV F complex and R4.C6-RSV F complex, we found partial overlap of their epitopes at RSV F site II. For instance, R4.C6 and hRSV14N4 shared common residues D263, P265, I266, N268, D269 and K272 in binding to antigenic site II, but with residues N262, T267 and K271 unique to hRSV14N4. The binding of these two antibodies to antigenic site II involves an angle difference of 150° and a spatial translation of 1.1 Å as estimated by DYNDOM [44] (Fig 4A). Furthermore, motavizumab and R4.C6 bind to site II at an angle difference of 117° and a spatial translation of 0.7 Å (Fig 4B). Their epitopes have common residues D263, N268, D269 and K272, with residues P265 and I266 unique to R4.C6 and residues N262, S275 and N276 unique to motavizumab.

In addition, we also compared our RSV F-R4.C6 structure with the crystal structure of site IV-specific mAb 101F with a 17-residue site IV peptide (PDB code: 3O45) [29] by superimposing on antigenic site IV residues N428 to N437. 101F specifically binds to RSV F antigenic site IV in the region of residues R429 to S436. The 101F epitope does not overlap with R4.C6 epitope (Fig 4C). The angle and spatial translation of R4.C6 in binding to RSV F differed from those of 101F by 162° and 12.8 Å, respectively. The unique binding angle of R4.C6 facilitates its interactions with both antigenic site II and site IV.

## Discussion

In this study, we reported the discovery of four new mAbs elicited in mice that were immunized with a near full-length RSV F NP (Table 1), followed by a series of detailed characterization of a novel mAb designated as R4.C6. Epitope binning by BLI using Octet QK384 instrument revealed that mAb R4.C6 broadly competed for binding with neutralizing humanized and human mAbs targeting antigenic site IIa (palivizumab, hRSV14N4 and R6.46) and site IIb (motavizumab and hRSV3J20) as well as non-neutralizing mAb (R6.29) binding to antigenic site VII. Unexpected, mAb R4.C6 also competed binding of neutralizing mAbs RSHZ19 and R1.42 that are directed at antigenic site IV. R4.C6 bound RSV F NP and a site II synthetic peptide with nanomole affinity (*K*_D_ = 0.07 and 12.5 nM, respectively), which were 7- and 30-fold higher than palivizumab (Table 1). However, the neutralization potency of R4.C6 was slightly lower than palivizumab (IC_50_ = 1078 vs. 323 ng/mL). Moreover, although R4.C6 had a similar binding affinity for RSV F NP as R1.42 (*K*_D_ = 0.07 vs. 0.04 nM), R4.C6 exhibited about 50-fold lower neutralization activity than R1.42 (IC_50_ = 1078 vs. 19.6 ng/mL). Therefore, despite a clear correlation of binding affinity and neutralization activity between palivizumab and its affinity-matured variant motavizumab (Table 1) [31], this correlation was not always observed in other mAbs. Probably other factors, including but not limited to, the accessibility of the target epitope(s) on the tightly packed RSV F glycoproteins on the viral surface are also in play. More structural and functional studies of RSV F-specific antibodies like the one reported here are urgently needed for a better understanding of mAb-mediated neutralization of RSV virus.

R4.C6 is unique in its ability to bind to both antigenic site II and site IV of RSV F glycoprotein. Single particle cryo-EM and 3D reconstruction of RSV F710-R4.C6 complex unraveled the structural basis of R4.C6 in recognizing this novel epitope on post-fusion RSV F. Three R4.C6 were found to be in association with a single RSV F trimer. The structure demonstrated that R4.C6 recognizes a quaternary epitope that involved two protomers of the RSV F trimer. Fine mapping demonstrated that two HCDRs and two LCDRs of R4.C6 interacted with antigenic sites II and IV. A comparison of binding poses to RSV F post-fusion for R4.C6 with hRSV14N4 (site IIa), motavizumab (site IIb), and 101F (site IV) clearly showed that R4.C6 binds to RSV F at an angle distinct from these antibodies (Fig 4).

In summary, the RSV F glycoprotein is a major candidate for vaccine development. However, it is structurally complex with multiple conformations and numerous antigenic epitopes. The structures of the RSV F in the pre-fusion and post-fusion conformations in complex with various mAbs have provided considerable insights into fine mapping of antigenic determinants in various conformations. In this report, we have used single particle cryo-EM and 3D reconstruction to determine the structural basis for the interaction of R4.C6 with a previously unknown epitope that encompasses antigenic sites II and IV on the post-fusion RSV F trimer at 3.9 Å resolution. The discovery and detailed characterization of the new R4.C6 epitope significantly deepen our understanding of the antigenic complexity of the F protein. Furthermore, the comparison of R4.C6 with a panel of other mAbs in terms of binding affinity and neutralization activity highlighted our incomplete understanding of structural and environmental factors that may affect the *in vitro* and *in vivo* activities of mAbs. More detailed characterization of RSV F-specific antibodies along this line is needed for a better understanding of mAbs directing at RSV virus.

## Materials and methods

### Cell lines, viruses, antibody reagents and synthetic peptides

HEp-2 cells (ATCC, CCL-23) were maintained in MEM with Earle’s salts, L-glutamine (Gibco Laboratories, Gaithersburg, MD, USA), 5% fetal bovine serum (FBS, Hyclone, Logan UT, USA) and antibiotics (Life Technologies, Grand Island, NY, USA). *Spodoptera frugiperda* (Sf9) insect cells (Invitrogen, Grand Island, NY, USA) were maintained in serum free medium as suspension cultures. *Trichoplusia ni* High Five cells (BTI-TN-5B1-4, ATCC CRL-10859) were maintained in Insect-XPRESS Medium with L-glutamine and antibiotics (Lonza, Walkersville, MD, USA). RSV/A Long (ATCC, VR-26) reference strain was obtained from ATCC (Manassas, VA, USA). Virus stock was prepared from clarified supernatants and stored at -80°C in PBS with 25% sucrose as a cryo-protectant. Palivizumab (Synagis) was obtained from MedImmune, Inc. (Gaithersburg, MD, USA) and motavizumab from National Institute of Standards and Technology (Gaithersburg, MD, USA). D25 [17] (Creative Biolabs, Shirley, NY, USA) and RSHZ19 [45] (Absolute Antibody, Oxford, UK) were purchased commercially. RSV14N4 and RSV3J20 [30] were kindly provided by Dr. J. Crowe (Vanderbilt University, Nashville, TN, USA). Palivizumab site II synthetic peptide and its various truncations were commercially synthesized by Peptide 2.0 (Chantilly, VA, USA).

### Expression and purification of RSV F NP, pre-fusion F and post-fusion F

RSV F DNA constructs were synthesized according to the RSV/A2 F0 gene sequence encoding residues 26–574 (Genebank accession number U63644) with gp64 signal peptide (SP) added to the 5’ end of all constructs and codon optimized for insect cell expression (GeneArt, Regensburg, DE). RSV F NP contained the intact C-terminal transmembrane (TM) and cytoplasmic tail (CT) domains, a mutant furin cleavage site II (KKRKRR to KKQKQQ) and a 10 amino-acid deletion (ΔF137—V146) in the hydrophobic fusion peptide (FP) [38, 46]. Pre-fusion F contained the intact TM/CT, a mutant furin site I (RARR to RARQ), deletion of the full p27 region (Δp27), ΔF137-V146 in the FP and harbored three stabilizing amino-acid substitutions N67I, S215P, and E487Q [47]. Post-fusion F was constructed by replacing the TM/CT domains with a C-terminal 6-histidine tag [35]. Pre-fusion F gene was cloned into Bac I baculovirus transfer vector (EMD Millipore), RSV F NP and post-fusion F genes were cloned into pFastBac 1 (Invitrogen), downstream of the *AcMNPV* polyhedron promoter. The recombinant baculovirus (BV) with the pre-fusion F gene was generated using the FlashBacGOLD BV system and those with RSV F NP and post-fusion F genes were generated using the Bac-to-Bac baculovirus system. For RSV F NP and pre-fusion F, BV-infected Sf9 cells were harvested by centrifugation and the cell pellets were extracted. Cell lysates were clarified and purified as previously described [38, 46]. Recombinant RSV F NP with TM/CT domains extracted from insect cell membranes were assembled into nanoparticles with morphology consistent with F oligomers [38]. For post-fusion F, BV-infected Sf9 cells were cultured for ~65 h at 27°C, and supernatants were collected by centrifugation (4000 × g). Post-fusion F was purified with an immobilized metal affinity column (IMAC) and ion exchange chromatography. These RSV F proteins were used to obtain the data reported in Table 1, Fig 1B and 1C, S1 and S2 Figs.

### Expression and purification of RSV F710 post-fusion

The RSV F710 was derived from the RSV F NP construct with the TM/CT domains replaced with the T4 fibritin foldon trimerization motif (GSGYIPEAPRDGQAYVRKDGEWVLLSTFL) and a 6-histidine tag added at the C-terminus of the F1 fragment [48, 49]. The gene was cloned into the BamH I/Hind III site of pFastBac DUAL vector (Thermo Fisher, Waltham, MA, USA) downstream of the AcMNPV polyhedron promoter. In addition, the Sf9 furin sequence (Genbank Accession No. CAA93116.1) was cloned between XmaI/KpnI site of the same pFastBac DUAL vector downstream of AcMNPV p10 promoter. Recombinant bacmid DNA was extracted from *E*.*coli* and transfected into Sf9 cells using Cellfectin II (Invitrogen, Carlsbad, CA, USA). The transfection supernatants were harvested and recombinant BVs plaque purified and amplified. Cultures of High Five cells at 1.5 × 10^6^ cells/mL were infected with the recombinant BV harboring the RSV F710 gene at multiplicity of infection = 2 and then incubated at 27°C for 48–60 hours. Cell culture supernatant containing secreted RSV F710 was collected by centrifugation for 30 minutes at 4,000 × g. RSV F710 glycoprotein was purified by binding to IMAC and eluted with 250 mM imidazole. The protein was further purified by ion exchange chromatography (Mono S 5/50 GL, GE Healthcare, USA) and size exclusion chromatography (Superdex 200 10/300 GL, GE Healthcare, USA) in buffer containing 20 mM Hepes, pH 7.0 and 150 mM NaCl. This protein was used in cryo-EM study as well as in Fig 1A and S3 Fig.

### Generation of mAbs

RSV F-specific mAbs were generated using modified standard methods [37]. Briefly, female BALB/c mice were immunized by intraperitoneal injection with 5 μg RSV F NP spaced two weeks apart. Four days after boosting, spleens were collected and single cell suspensions prepared with a homogenizer. Splenocytes were pooled and depleted of IgM B lymphocytes with a magnetic cell sorting system (Miltenyi Biotec, Auburn, CA, USA). IgG-enriched splenocytes were fused with P3X63.Ag.6.5.3 myeloma cells [37]. Hybridomas were screened by RSV F ELISA and positive cultures cloned by limiting dilution. Hybridoma cell lines were expanded in 75 cm^2^ T-flasks in serum free media.

The mice protocol was approved by A&G IACUC committee (Animal Welfare Assurance Number D16-00700) with the Approval Number JH-01. Mice were euthanized by CO_2_ inhalation followed by cervical dislocation. CO_2_ inhalation was performed using a gradual fill method with a displacement rate from 10% to 30% of the chamber volume/min. In order to reduce unnecessary stress, mice were euthanized in their cage when possible. A total of three athymic nude mice (Charles River, Wilmington, MA) were used in the study.

### Virus neutralization assay

The virus neutralization activity of the mAbs was determined with a micro-neutralization assay. Briefly, purified mAbs were serially diluted over the concentration range of 0.004–10 μg/mL in 96-well tissue culture plates and mixed with 200–350 TCID_50_ (50% tissue culture infectivity dose) of RSV/A Long for 2 hours at 37°C. Low passage HEp-2 cells (2.5 x 10^4^ cells) were added to the antibody/virus mixture at 37°C in 5% CO_2_ incubator for 4–5 days. The plates were washed and fixed. Infectious virus was detected by adding optimally diluted mouse anti-RSV M2-1 (clone RSV 5H5) mAb (Novus Biologicals, LLC, Littleton, CO, USA). The plates were washed, horseradish peroxidase (HRP) goat anti-mouse IgG added, followed by addition of TMB (3,3',5,5'-tetramethylbenzidine) substrate. The half maximal inhibitory concentration (IC_50_) was determined by a 4-parameter curve fitting using Prism software.

### Epitope binning by BLI

Antibody cross-competition was performed by BLI using an Octet QK384 system (Pall Forte Bio, Fremont, CA, USA). Histidine tagged RSV F710 protein (10 μg/mL) was immobilized on anti-penta-histidine biosensor tips. Captured RSV F710 was exposed to mAbs in two sequential steps. Biosenor tips were exposed to the first mAb (20 μg/mL) for 5 minutes followed by dipping the tips in the second analyte antibody (10 μg/mL) for an additional 5 minutes. Assays were performed at 30°C with continuous agitation at 1,000 rpm. If RSV F protein binding by the first mAb prevented or reduced the binding of the competing second mAb, the competing mAb was considered to bind to similar epitopes. Conversely, if the first mAb did not interfere with binding of the second mAb, the antibodies were considered to bind to distinct epitopes. The percent (%) inhibition of antibody binding by the competing mAbs was calculated with Octet data analysis HT10.0 software by the following formulation: % inhibition = 100 –[analyte mAb binding in presence of competitor mAb / binding of analyte mAb alone] x 100.

### MAb binding to RSV F proteins by BLI

Antigenic site-specific mAb binding to RSV F NP vaccine, pre-fusion, and post-fusion conformers was determined by BLI using an Octet OK384 instrument (Pall FortéBio, Fremont, CA, USA). Anti-human Fc BLI biosensor tips were used to immobilize mAbs D25 (site Ø), palivizumab (site IIa), RSHZ19 (site IV) and anti-mouse Fc biosensor tips were used to immobilize R1.42 and R4.C6. Tips were exposed to mAbs (10 μg/mL) for ~600 seconds and equilibrated to baseline in equilibration buffer (Pall FortéBio) for 60 seconds. The coated tips were transferred to wells containing 20 μg/mL RSV F NP vaccine, post -fusion F or pre-fusion F and allowed to associate for 600 seconds followed by dissociation for 400 seconds. The percent antibody binding (% Binding) to RSV F was analyzed with Octet HT 10.0 software with reference to the buffer control.

### MAb binding affinity by surface plasmon resonance

Surface plasmon resonance (SPR) was used to assess the binding affinity of mAbs to RSV F NP, pre-fusion F, post-fusion F or various synthetic peptides derived from RSV F antigenic site II sequence (254-NSELLSLINDMPITNDQKKLMSNNV-278) using a Biacore T200 instrument (GE Healthcare, Baltimore, MD). Protein A or protein G (Thermo Fisher Scientific, Waltham, MA) was immobilized on CM5 chips through amine-coupling reaction. The targeted coupling level was greater than 3000 response units (RU). All antibodies were diluted to 2 μg/mL and injected over the protein A or protein G immobilized chip at approximately 10 μL/min for 45 seconds for RSV F binding kinetics assay or for 90 seconds for antigenic site II peptide binding kinetics assay. RSV F was injected at increasing concentrations (3.3, 10, 30, 90 nM) over the antibody immobilized chip at 40 μL/min for 180 seconds followed by 600 seconds dissociation time. Similarly, site II peptides were injected at increasing concentration (100, 200, 400, 800 nM) for 180 seconds followed by 600 seconds buffer flow. The sensorgrams were analyzed by Biacore kinetics analysis using a 1:1 fit model to determine the *k*_a_ and *k*_d_ rates. The apparent equilibrium constant *K*_D_ was calculated using the equation *K*_D_ =   *k*_d_/*k*_a_. Chips were regenerated by injection of 100 mM HCl at 40 μL/min for 45 seconds.

### R4.C6 purification and Fab preparation

To produce R4.C6 Fab, the hybridoma was grown in serum free medium in a 7 L bioreactor (Southern Biotech, Birmingham, AL, USA). The cleared supernatant was concentrated and applied to a protein-G affinity column following the manufacturer’s protocol (GE Healthcare Life Sciences). Purified R4.C6 was digested with papain (Pierce Fab Preparation Kit, Thermo Fisher Scientific) with mild reduction. Antigen-binding fragments (Fab) were purified from undigested IgG and Fc fragments by ion exchange chromatography (Southern Biotech, Birmingham, AL, USA). The Fab was 98.8% pure as determined by reverse phase HPLC.

### Preparation of the R4.C6 Fab and RSV F complex

To prepare R4.C6 Fab in complex with RSV F protein, purified RSV F710 trimer was mixed with R4.C6 at molar ratio of 1:4. This ratio ensured the RSV F protein was saturated by R4.C6. The mixture was put on ice for 30 minutes and then purified on a Superdex 200 10/300 GL gel filtration column (GE Healthcare, USA) with a running buffer of 10 mM Hepes, 100 mM NaCl, pH 7.0. The complex was eluted at a volume of 9.8 mL, and the excess R4.C6 was eluted at 16 mL. Compared with the size-exclusion chromatography profile of RSV F710 protein alone, the R4.C6-RSV F complex peak had a 1.5 mL shift in elution volume. Samples from each peak were analyzed by using 12% reduced Bis-Tris SDS-PAGE gel. The eluted complex was concentrated to 2 mg/mL for storage and for preparing the cryo-EM specimen.

### Preparation of graphene-oxide-support-covered grids

Graphene oxide dispersion (Sigma-Aldrich, USA; 4 mg/mL in H_2_O) was diluted to 0.2 mg/mL with Milli-Q water (mqH_2_O) and centrifuged at 300 × g for 30 seconds to remove large aggregates. Quantifoil R1.2/1.3 200 mesh Cu holey grids (Quantifoil, Jena, Germany) were glow discharged for 75 seconds. 3 μL of the graphene oxide suspension was added to the carbon side of the grid and incubated for 2 minutes. After incubation, the graphene oxide solution was removed by brief blotting with Whatman No. 1 filter paper from the side, followed by washing with mqH_2_O and blotting. The coverage of graphene oxide on the grid was visualized using transmission electron microscope (TEM) at low magnification mode before use. The graphene-oxide-coated grids were used for plunge-freezing without any further treatment.

### Preparation of cryo-EM specimen

A 3.5 μL aliquot of the complex diluted to 0.05mg/mL was applied onto Quantifoil R1.2/1.3 200 mesh Cu holey grids coated with graphene oxide. After 15-second incubation, the grid was automatically blotted for 4 seconds from the specimen side with Whatman No. 1 filter paper and immediately plunged into liquid ethane using Leica EM GP automatic plunge freezer (Leica Microsystems, Vienna, Austria) with its environmental chamber set at 22°C and relative humidity at 98%. The grids were transferred and stored in liquid nitrogen before imaging.

### Cryo-EM data collection and image processing

All data were collected on a JEM3200FSC cryo-electron microscope (JEOL, Peabody, MA, USA) operated at 300kV, with energy slit of the in-column filter of 20 eV. Images were recorded using K2 Summit direct electron detector (DDD) camera (Gatan, Inc, Warrendale, PA, USA) in super-resolution electron counting mode at 30,000x microscope magnification (corresponding to a calibrated physical pixel size of 1.2546 Å). The dose rate is 5 electrons/Å^2^/sec and 50 frames were acquired in a total exposure time of 10 sec. A total of 2,734 DDD movie stacks were collected.

The dose-fractionated super-resolution raw image stacks were binned 2 X 2 by Fourier cropping resulting in a pixel size of 1.2546 Å for further image processing. Each image stack (containing all 50 frames) was subjected to motion correction using MotionCor2 [50]. Gctf [51] was used to estimate the contrast transfer function parameters. 7,456 particles were boxed out manually using ‘e2boxer.py’ in EMAN2 [52] as a particle subset to calculate reference-free 2D class averages, which was then used as templates for automated particle picking of the entire date set. 543,639 particles were picked finally. An initial map was generated with 3-fold symmetry from 2-D reference-free averages using EMAN2. This initial reference map was masked so that only the RSV F part was kept. It was then low pass filtered to 60 Å resolution and used as a starting model. RELION2.0 package [53] was used for subsequent imaging processing. The initial runs of 2D and 3D classifications were used to remove false positive particles from the auto-picking. Only those good particles were selected for further analysis. Several rounds of iterative 3D classification and 3D auto-refinement were performed. 234,479 particles were used in the final refinement to achieve a 3.9 Å resolution density map. A soft mask in RELION post-processing was applied before computing the FSCs. The final resolution was estimated using the gold standard of FSC = 0.143. The density map was sharpened by applying a *B*-factor of -250 Å^2^ estimated by an automated procedure. Local resolution variations were estimated with ResMap [54] using two independent maps.

### Model building, refinement and analysis of the RSV F-R4.C6 complex

The crystal structures of RSV F in pre-fusion or post-fusion conformation (PDB codes: 4JHW and 3RRR) were separately docked into the map using Chimera [39], where the post-fusion structure fitted much better. Since there is no known structure for R4.C6 Fab, a homology model was built using the known structures of heavy chain and light chain variable domains that display the highest sequence homology to R4.C6 (PDB code: 1I3G [55]). The molecular dynamics flexible fitting (MDFF) [56] method was used to flexibly fit the homology model into the cryo-EM map. The model for the complex was then refined using Phenix [40] real-space-refine followed by manually adjustment using Coot [41] to optimize the local fit into the density. In addition, Parallel Continuous Simulated Tempering (PCST)—assisted structural refinement (F.N., Q.W. and J.M., unpublished) was used. PCST algorithm is implemented in Gromacs program [57]. The structures without restraints during the simulation were extracted and ranked by GOAP potential [58]. The structure with the lowest GOAP score was then fitted into EM map by phenix.real_space_refine program. After PCST refinement, the model quality and geometry statistics as calculated by MolProbity were significantly improved. For structural analysis, the total buried surface area between R4.C6 and RSV F710 was calculated using AREAIMOL in CCP4 [59], and the relative orientation of different mAbs in binding to RSV F protein was estimated by DYNDOM [44] in CCP4 [59].

## Supporting information

S1 Fig Sensorgrams of SPR analysis of the binding affinity between a panel of mAbs and RSV F NP or site II peptide. RSV F NP of concentrations of 3.33, 10, 30, 90 nM or site II peptide of concentrations of 100, 200, 400, 800 nM were used (low to high). The black curves were the fitting curves. The x-axis is time (second) and y-axis is resonance unit (RU). The binding affinity values were reported in Table 1.
(TIF)

S2 Fig Sensorgrams of SPR analysis of the binding affinity between a panel of mAbs and RSV F NP vaccine, post-fusion trimer or pre-fusion trimer. RSV F of concentrations of 3.33, 10, 30, 90 nM were used (low to high). The x-axis is time (second) and y-axis is resonance unit (RU). The black curves were the fitting curves. The binding affinity values were reported in Fig 1C.
(TIF)

S3 Fig Purification of R4.C6 Fab in complex with RSV F710 glycoprotein. Size-exclusion chromatography profiles of RSV F-R4.C6 complex (black solid line) and RSV F trimer alone (blue dashed line) using Superdex 200 10/300 GL column (GE Healthcare). The peaks of RSV F-R4.C6 complex, RSV F trimer, and excess R4.C6 are labeled. Coomassie-stained 12% reduced Bis-Tris SDS-PAGE gel shows RSV F (F1 and F2) and R4.C6 Fab in the complex peak. Protein standards of known molecular weight are labeled.
(TIF)

S4 Fig Cryo-EM 3D reconstruction for RSV F-R4.C6 complex and resolution estimation. (A) Fourier power spectrum of the micrograph shown in Fig 2A with Thon rings and water ring 3.5 Å labeled. (B) Euler angle distribution plot of all particles used for the final 3D reconstruction. Bar length and color (blue, low; red, high) is proportional to the number of particles contributing to each specific view. Refined reconstruction map from different angles are also shown. (C) Cryo-EM map of R4.C6 Fv in complex with RSV F is colored according to ResMap local resolution estimation. The cryo-EM map exhibits local resolution ranging from 2.7 Å to 4.6 Å. (D) Gold-standard FSC curves for the 3D reconstruction (blue curve) generated with RELION2.0 and map *vs*. model (red curve), marked with resolution corresponding to FSC = 0.143.
(TIF)

S5 Fig Representative cryo-EM maps of the final RSV F-R4.C6 complex. (A-E) Residues of RSV F. (A) Helix in residues 78–95; (B) Helix in residues 227–239; (C) Helix in residues 205–227; (D) Residues 29–42; (E) Residues 403–417. (F-G) Residues of R4.C6. (F) Residues 66–81 of R4.C6 heavy chain. (G) Residues 62–76 of R4.C6 light chain. The cryo-EM map for each selected region is shown in black mesh and superimposed on the corresponding RSV F-R4.C6 complex model. Residue atoms colored as the following: C = cyan, N = blue, O = red, S = yellow.
(TIF)

S6 Fig Sensorgrams of SPR analysis of the binding affinity between site II peptide or its truncated variants and R4.C6 or Palivizumab. Site II peptides of concentrations of 100, 200, 400, 800 nM were used (low to high). The black curves were the fitting curves. The x-axis is time (second) and y-axis is resonance unit (RU). The binding affinity values were reported in Table 2.
(TIF)

S1 Table Cryo-EM data collection and refinement statistics. (DOCX)
