# Supplementary figures and images for: Structure basis of neutralization by a novel site II/IV antibody against respiratory syncytial virus fusion protein
Source: PLoS One. 2019 Feb 7;14(2):e0210749. doi: 10.1371/journal.pone.0210749 (PMC6366758; doi:10.1371/journal.pone.0210749)

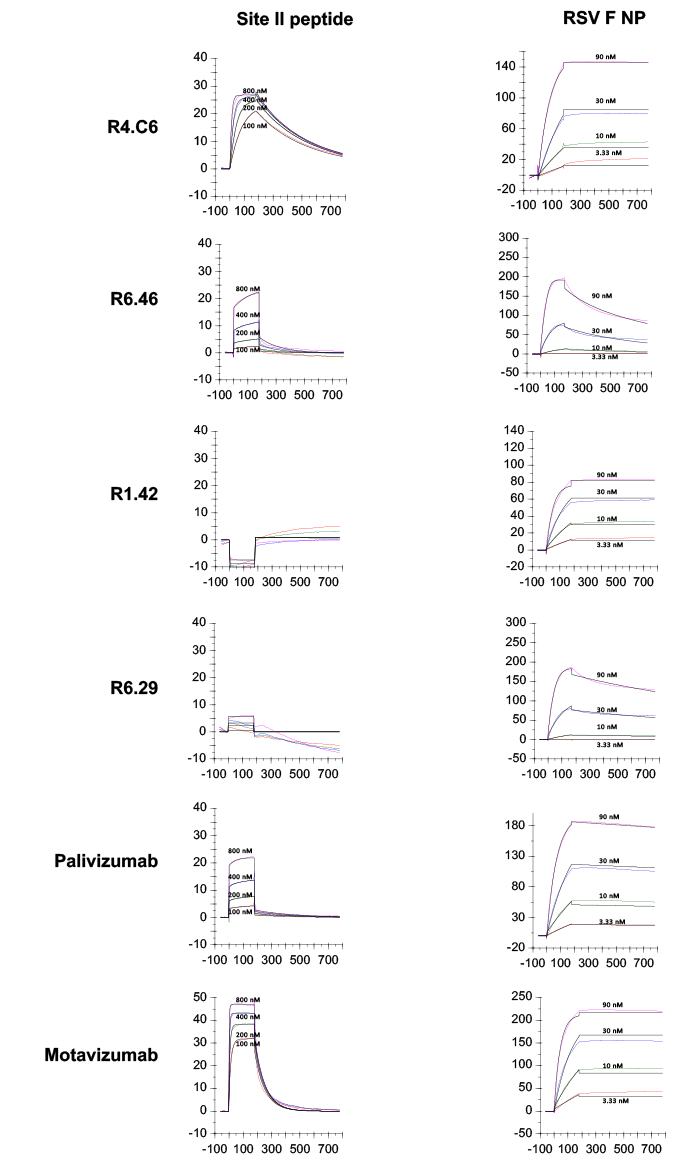

Supplement: S1 Fig — RSV F NP of concentrations of 3.33, 10, 30, 90 nM or site II peptide of concentrations of 100, 200, 400, 800 nM were used (low to high). The black curves were the fitting curves. The x-axis is time (second) and y-axis is resonance unit (RU). The binding affinity values were reported in Table 1. (TIF) [file pone.0210749.s001.tif]

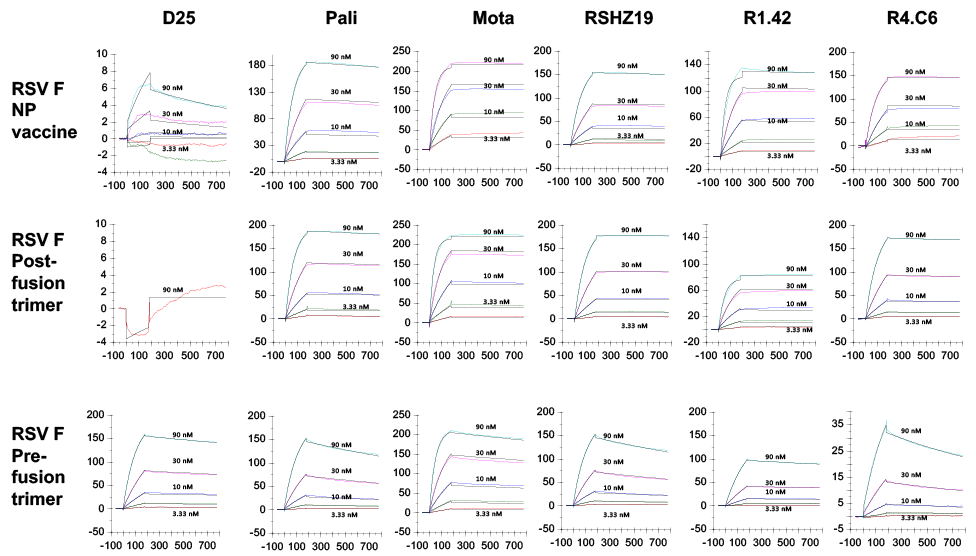

Supplement: S2 Fig — RSV F of concentrations of 3.33, 10, 30, 90 nM were used (low to high). The x-axis is time (second) and y-axis is resonance unit (RU). The black curves were the fitting curves. The binding affinity values were reported in Fig 1C. (TIF) [file pone.0210749.s002.tif]

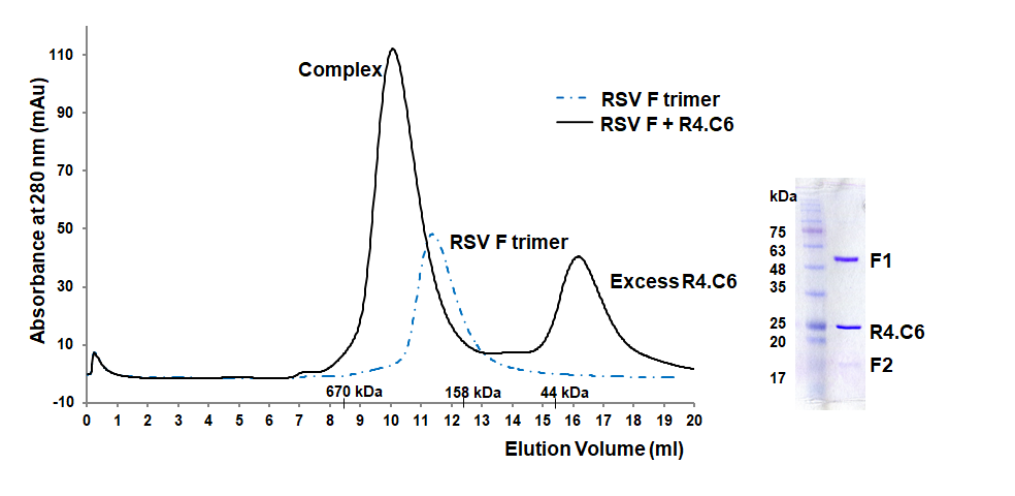

Supplement: S3 Fig — Size-exclusion chromatography profiles of RSV F-R4.C6 complex (black solid line) and RSV F trimer alone (blue dashed line) using Superdex 200 10/300 GL column (GE Healthcare). The peaks of RSV F-R4.C6 complex, RSV F trimer, and excess R4.C6 are labeled. Coomassie-stained 12% reduced Bis-Tris SDS-PAGE gel shows RSV F (F1 and F2) and R4.C6 Fab in the complex peak. Protein standards of known molecular weight are labeled. (TIF) [file pone.0210749.s003.tif]

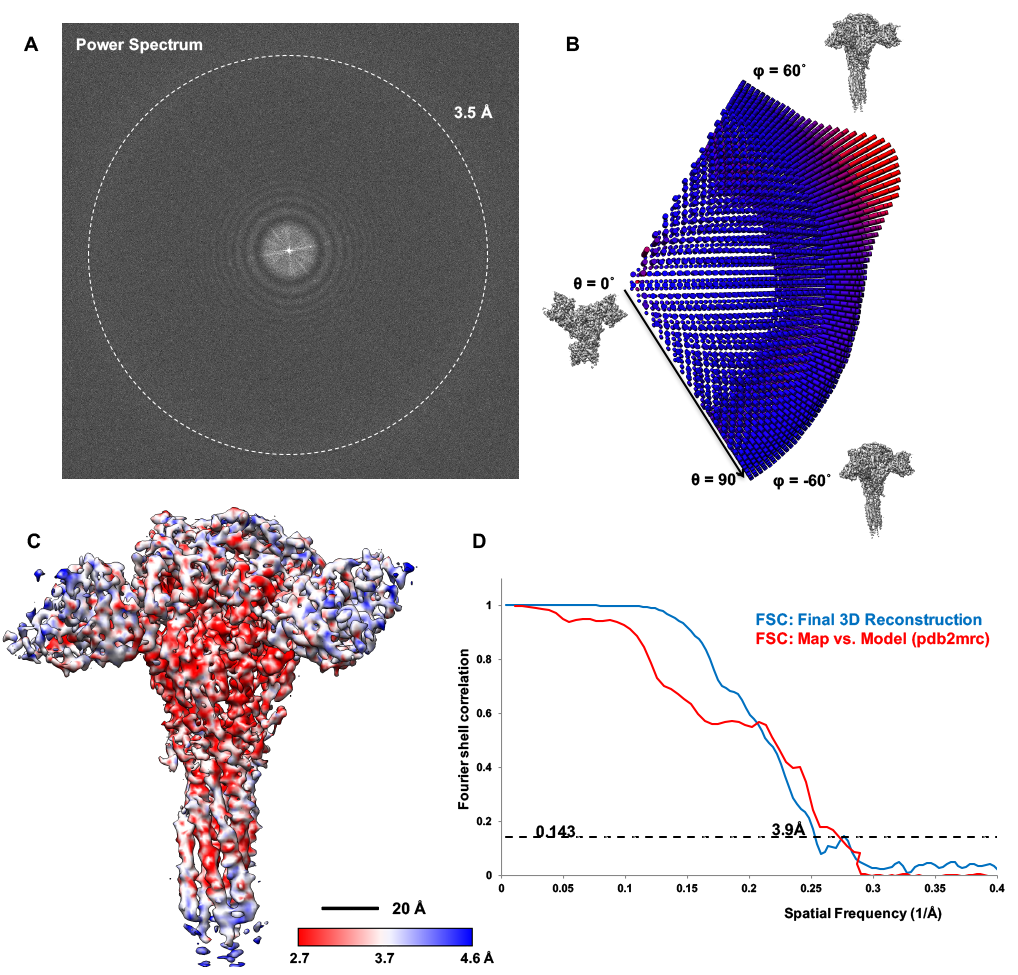

Supplement: S4 Fig — (A) Fourier power spectrum of the micrograph shown in Fig 2A with Thon rings and water ring 3.5 Å labeled. (B) Euler angle distribution plot of all particles used for the final 3D reconstruction. Bar length and color (blue, low; red, high) is proportional to the number of particles contributing to each specific view. Refined reconstruction map from different angles are also shown. (C) Cryo-EM map of R4.C6 Fv in complex with RSV F is colored according to ResMap local resolution estimation. The cryo-EM map exhibits local resolution ranging from 2.7 Å to 4.6 Å. (D) Gold-standard FSC curves for the 3D reconstruction (blue curve) generated with RELION2.0 and map vs. model (red curve), marked with resolution corresponding to FSC = 0.143. (TIF) [file pone.0210749.s004.tif]

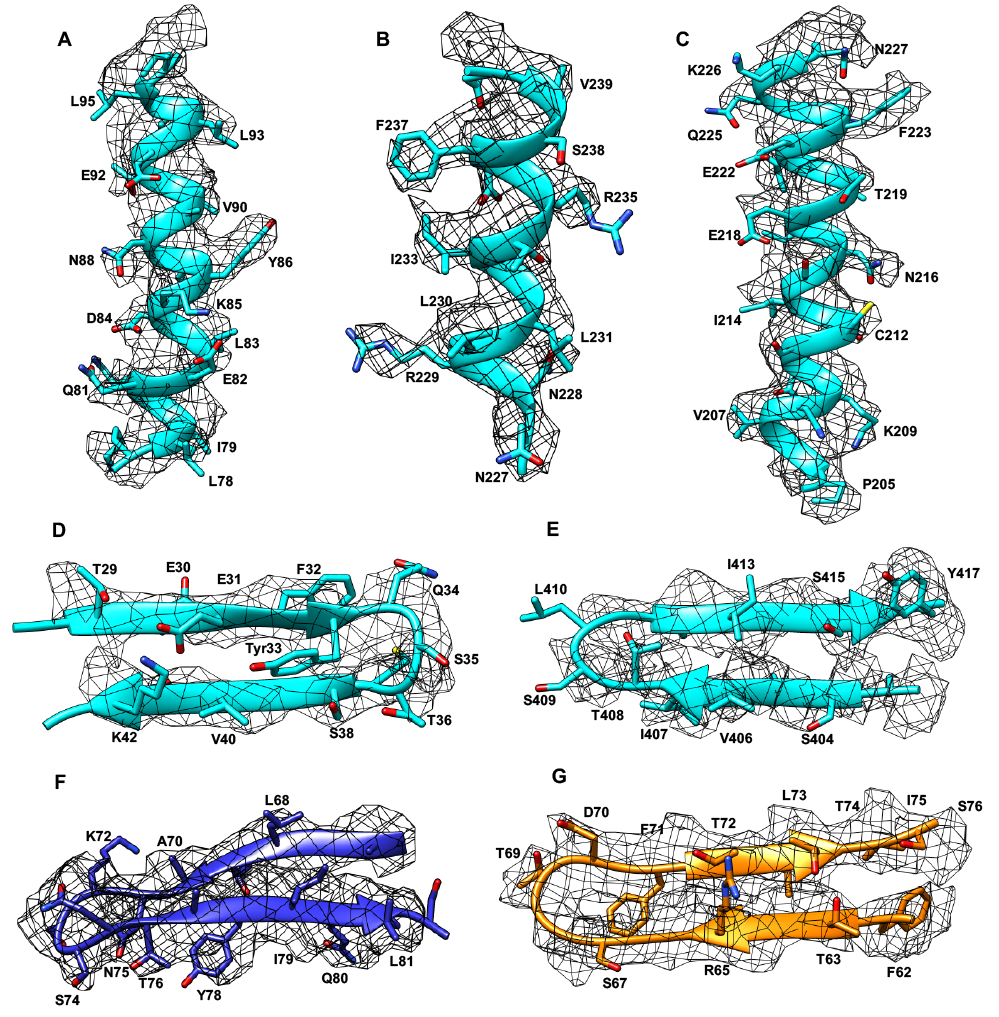

Supplement: S5 Fig — (A-E) Residues of RSV F. (A) Helix in residues 78–95; (B) Helix in residues 227–239; (C) Helix in residues 205–227; (D) Residues 29–42; (E) Residues 403–417. (F-G) Residues of R4.C6. (F) Residues 66–81 of R4.C6 heavy chain. (G) Residues 62–76 of R4.C6 light chain. The cryo-EM map for each selected region is shown in black mesh and superimposed on the corresponding RSV F-R4.C6 complex model. Residue atoms colored as the following: C = cyan, N = blue, O = red, S = yellow. (TIF) [file pone.0210749.s005.tif]

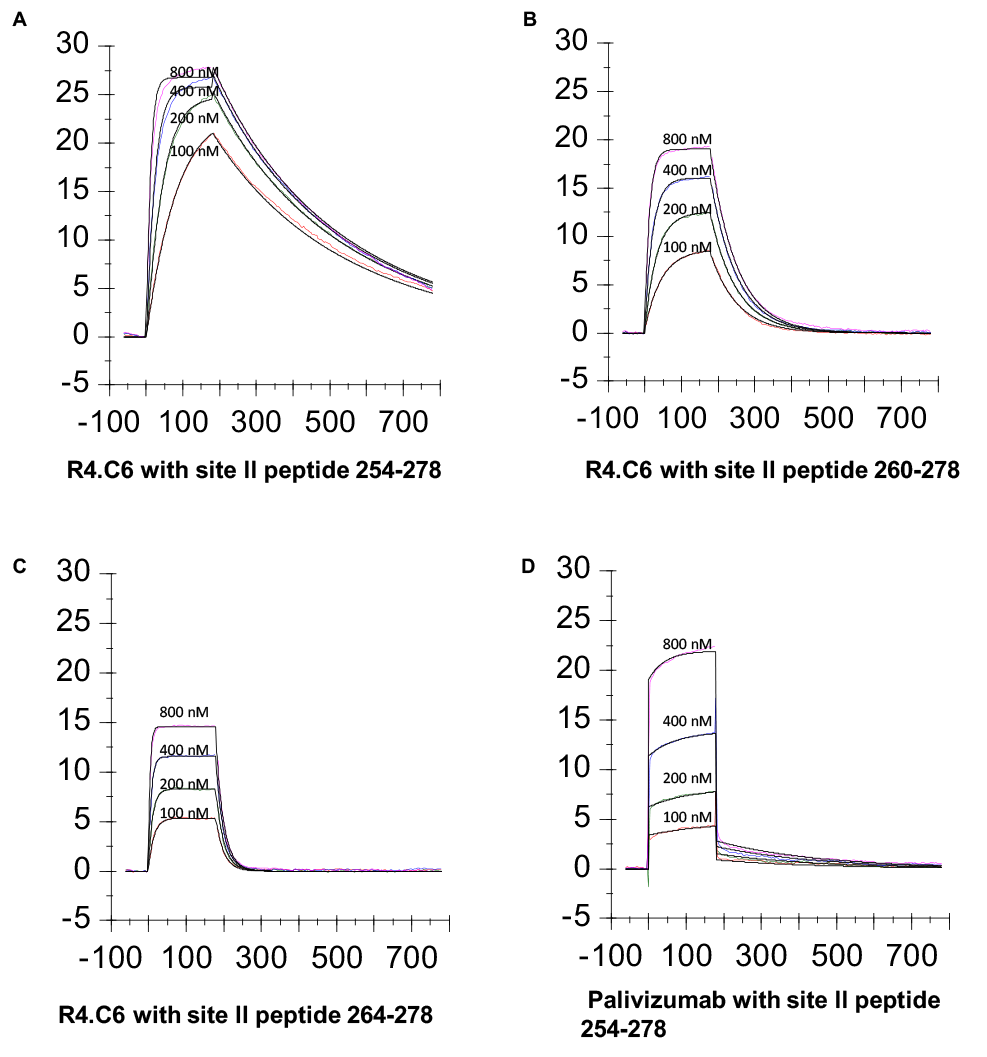

Supplement: S6 Fig — Site II peptides of concentrations of 100, 200, 400, 800 nM were used (low to high). The black curves were the fitting curves. The x-axis is time (second) and y-axis is resonance unit (RU). The binding affinity values were reported in Table 2. (TIF) [file pone.0210749.s006.tif]
