# Supplementary material for: Structure basis of neutralization by a novel site II/IV antibody against respiratory syncytial virus fusion protein
Source: PLoS One. 2019 Feb 7;14(2):e0210749. doi: 10.1371/journal.pone.0210749 (PMC6366758; doi:10.1371/journal.pone.0210749)
Supplement: S1 Table — (DOCX) [file pone.0210749.s007.docx]

**S1 Table. Cryo-EM data collection and refinement statistics**

| Data Collection |  |
| --- | --- |
| Microscope | JEM3200 |
| Voltage (kV) | 300 |
| Camera Detector | Gatan K2 Summit |
| Exposure time (s) | 10 |
| Dose rate (e^-^ / Å^2^/s) | 5 |
| Total electron dose (e^-^ /Å^2^) | 50 |
| Pixel size (Å) | 1.2546 |
| Reconstruction Software | Relion2.0 |
| Number of micrographs | 2734 |
| Number of particles (processed) | 543,639 |
| Number of particles (in final map) | 234,479 |
| Symmetry | C3 |
| Map-sharpening *B* factor (Å^2^) | -250 |
| Final resolution (Å) | 3.9 |
|  |  |
| Refinement |  |
| Resolution (Å) | 3.9 |
| CC (Around atoms) | 0.7161 |
| FSC (Model/Map Around atoms) | 0.9114 |
| R.m.s. deviation |  |
| Bond length (Å) | 0.014 |
| Bond angle (˚) | 1.073 |
| Validation |  |
| MolProbity score | 1.97 |
| Clashscore, all atoms | 9.81 |
| Rotamer outliers (%) | 0.30 |
| Ramachandran plot |  |
| Favored (%) | 93.48 |
| Allowed (%) | 6.52 |
| Outliers (%) | 0 |
